# Using Phylogenetic, Functional and Trait Diversity to Understand Patterns of Plant Community Productivity

**DOI:** 10.1371/journal.pone.0005695

**Published:** 2009-05-27

**Authors:** Marc W. Cadotte, Jeannine Cavender-Bares, David Tilman, Todd H. Oakley

**Affiliations:** 1 National Center for Ecological Analysis and Synthesis, University of California Santa Barbara, Santa Barbara, California, United States of America; 2 Department of Biological Sciences, University of Toronto - Scarborough, Scarborough, Ontario, Canada; 3 Department of Ecology, Evolution and Behavior, University of Minnesota, St. Paul, Minnesota, United States of America; 4 Department of Ecology, Evolution and Marine Biology, University of California Santa Barbara, Santa Barbara, California, United States of America; University of Sheffield, United Kingdom

## Abstract

**Background:**

Two decades of research showing that increasing plant diversity results in greater community productivity has been predicated on greater functional diversity allowing access to more of the total available resources. Thus, understanding phenotypic attributes that allow species to partition resources is fundamentally important to explaining diversity-productivity relationships.

**Methodology/Principal Findings:**

Here we use data from a long-term experiment (Cedar Creek, MN) and compare the extent to which productivity is explained by seven types of community metrics of functional variation: 1) species richness, 2) variation in 10 individual traits, 3) functional group richness, 4) a distance-based measure of functional diversity, 5) a hierarchical multivariate clustering method, 6) a nonmetric multidimensional scaling approach, and 7) a phylogenetic diversity measure, summing phylogenetic branch lengths connecting community members together and may be a surrogate for ecological differences. Although most of these diversity measures provided significant explanations of variation in productivity, the presence of a nitrogen fixer and phylogenetic diversity were the two best explanatory variables. Further, a statistical model that included the presence of a nitrogen fixer, seed weight and phylogenetic diversity was a better explanation of community productivity than other models.

**Conclusions:**

Evolutionary relationships among species appear to explain patterns of grassland productivity. Further, these results reveal that functional differences among species involve a complex suite of traits and that perhaps phylogenetic relationships provide a better measure of the diversity among species that contributes to productivity than individual or small groups of traits.

## Introduction

For nearly two decades, researchers have tested the prediction that community productivity is positively related to plant diversity [1]–[6]. One guiding assumption has been that greater diversity in functional traits allows species to access more of the total resources [7]–[9], whether they be nutrients, water, pollinators or fungal symbionts, and allows multiple competing species to coexist [10]. Researchers have advocated measuring diversity in functional attributes relevant to those critical limiting resources and assumed that this should be the best predictor of community productivity and ecosystem functioning [11]–[13]. However, identifying the critical resources over pertinent temporal and spatial scales as well as the most relevant functional traits can be challenging.

The first approximation to classifying critical functional differences has been to group species into functional groups, which is often based on broad morphological and physiological similarities (e.g., C4, C3, legumes, etc.). The richness of functional groups has been used to potentially explain variation in community productivity [7], [13], [14]. But functional group richness is a problematic measure for two reasons. First, the removal or addition of “functionally redundant” species may have effects on community dynamics and processes [15]–[17], indicating that there are important functional differences not captured by broad groupings. Competition theory suggests there must be some niche differentiation, no matter how similar species are to stably coexist. The second reason is that functional group richness tends to predict only a limited amount of variation in productivity [18] and may even explain less variation than having randomly assigned groups [19].

Given that functional groups may be inadequate representations of critical functional diversity, ecologists have sought other ways of measuring functional diversity by measuring specific *a priori* selected traits [e.g.], [20], [21,22]. In contrast to measuring specific traits, other measures of functional diversity use multivariate techniques to evaluate trait differences/similarities among species without reliance on a small number of traits selected *a priori*. The first multivariate strategy, functional attribute diversity (FAD), introduced by Walker and colleagues [23], sums species distances in trait space as a measure of total trait or functional dissimilarity. The second strategy, functional diversity (FD) by Petchey and Gaston [24] essentially uses the FAD trait distance matrix to create a functional dendrogram from a clustering routine. FD then corresponds to the total dendrogram branch lengths connecting community members together. In a comparison of FAD and FD, Petchey et al. [25] show that FD better explains variation in community biomass accumulation. The final multivariate strategy we introduce here is a variation of the FD strategy. Since FD and FAD do not account for groups of correlated traits, we use nonmetric multidimensional scaling (NMDS) to create the distance matrix that accounts for correlated traits. Then we perform the FD clustering to produce branch lengths.

All these multivariate techniques, while relaxed from strict *a priori* trait decisions, may still be sensitive to which traits are included in the analyses [25]. Further, multivariate distances reflect the magnitude of scale units used (e.g., cm vs. m) and the variation in scale for different traits as opposed to the actual difference in ecological function (e.g., does a 10% difference in leaf size have the same ecological consequences as a 10% difference in seed mass?). Given these potential limitations of trait-based approaches, we advocate the use of another metric, phylogenetic diversity (PD) –that is the sum of phylogenetic branches connecting species together. If phenotypic dissimilarity is correlated with evolutionary divergence times [26]–[28], then the more divergent two species are, the greater likelihood that they differ ecologically. As a diversity measure, PD has been shown for some datasets to better explain variation in community productivity than species or functional group richness [9], [18].

In this paper, we compare the efficacy of explaining variation in community productivity with seven different types of trait diversity measures: 1) species richness, 2) variation in 10 individual traits, 3) functional group richness, 4) Walker and colleagues' (1999) Functional Attribute Diversity (FAD), 5) Petchey and Gaston's (2002) Functional Diversity (FD), 6) our Nonmetric Multi-Dimensional Scaling (NMDS) approach, and 7) community PD. Our goal is to find which of these various diversity measures provides the best possible explanation of patterns of community productivity, moving from simple, single-variable models to metrics that represent full community trait differences.

## Methods

### Study site

**Figure 1:**
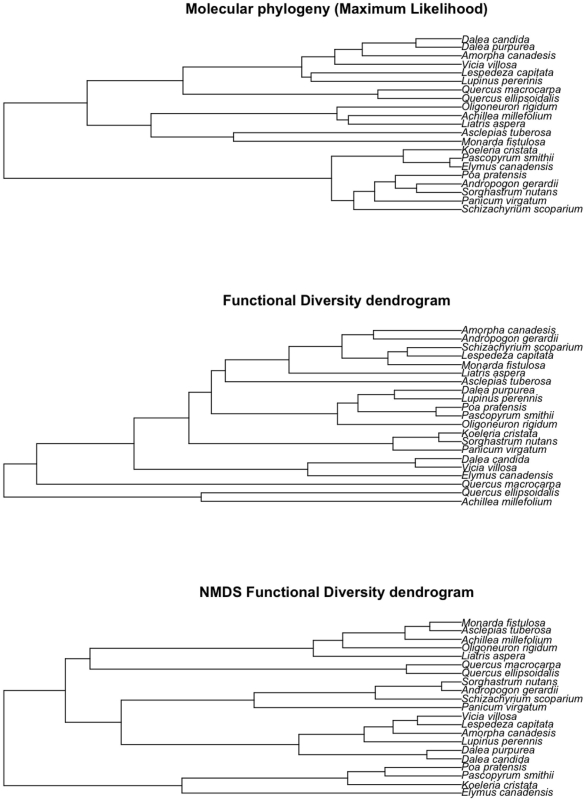
Three dendrograms representing relationships among species. The first is based on maximum likelihood analyses of genetic sequences from four genes. The second uses the functional diversity methodology of Petchey and Gaston (2002) on all measured traits. The third dendrogram also uses the functional diversity method on three orthogonal dimensions from nonmetric multi-dimensional scaling.

In 1993, the vegetation and seed bank in a post-abandonment agricultural field located at Cedar Creek Natural History Area, Minnesota, USA, were removed via herbicide, burn and bulldozing treatments. The following year 13×13 m plots were seeded with 1, 2, 4, 8 or 16 grassland savanna species (experiment 120). Plot composition was randomly chosen from a pool of 18 species that included four C3 grasses, C4 grasses, legumes, non-legume herbaceous forbs and two woody species. At each level of diversity 28–35 replicates were established, and plot composition was maintained by manually weeding and annual burns [for more details see: http://www.cedarcreek.umn.edu/research/exper/e120/, [2], [13], [29]]. In 1995 three more species were added to substitute for poorly germinating species from the original 18, but subsequent weeding did not target the poor germinators meaning that 21 species were actually included in this experiment (species are identified in Fig. 1).

As an estimate of productivity, peak aboveground biomass was measured by clipping, drying and weighing four 0.1 m by 3.0 m strips per plot. Biomass was sampled annually from 1996 to 2007 and we here use the average in biomass production as our dependent variable. The long-term average biomass was used because inter-annual variation was due largely to subsampling variance and regional climatic variation [30], [31]. In plots that carried fire the oaks (*Quercus ellipsoidalis* and *Q. macrocarpa*) performed poorly –contributing little to productivity, and so they were excluded from the calculation of the trait, functional and phylogenetic diversity metrics.

### Phylogeny construction

We constructed a phylogeny for the species used in this experiment and a second biodiversity experiment at Cedar Creek (i.e., Experiments 120 and 123), which included a total of 31 species (see Appendix S1 for a list of all species). In February, 2008, for each of the 31 species, we searched GenBank [32] for four gene sequences commonly used in published angiosperm phylogenies : *matK, rbcl, ITS1* and *5.8s*. Of the 31 species, 14 had at least one gene represented in Genbank and for a further 16 species, we used gene sequences from a congeneric relative not included in these experiments. Collectively, the species used in this experiment represent many of the deep historical angiosperm bifurcations, relative to the number of branches connecting close relatives. Therefore, the effect on branch length estimates from using congeneric species is likely minimal, so long as congenerics are monophyletic with the species used in the experiment. We also included two representatives of early diverging angiosperm lineages as outgroup species, *Amborella trichopoda* and *Magnolia grandiflora*, and we added 4 other species, each represented by multiple genes, that were included in clades whose members did not have overlapping sequences (see Appendix S1). For these 36 species we aligned sequences using MUSCLE [33]. We then selected best-fit maximum likelihood models of nucleotide substitution for each gene using the Akaike Information Criterion, as implemented in Modeltest [34], [35].

Using the aligned sequences and the best-fit models of nucleotide substitution, we estimated a maximum likelihood phylogeny using the PHYML algorithm with a BIONJ starting tree [36], [37]. To assess nodal support on maximum likelihood phylogenies, we report Approximate Likelihood Ratio Test (aLRT) scores, which have been shown to correlate with ML bootstrap scores, but require much less computational time [37]. The maximum likelihood tree is available in Appendix S1. A single species that lacked any genetic data, *Rudbeckia hirta*, was added as a polytomy with *Liatris aspera* and *Coreopsis palmata* because they are all considered members of the Asteroideae subfamily (see Appendix S1). For the analyses in the present paper, we pruned out the 10 species not used in experiment 120 (Fig. 1 shows the pruned phylogeny and Figure 1 in Appendix S1 shows the full phylogeny for both experiments). We did not rerun PHYML on the subtree members due to the sparseness of the gene matrix, especially for the Asteraceae species.

From the phylogeny, we calculated phylogenetic diversity (PD) for each experimental plot as the total phylogenetic branch lengths connecting only the community members together not including the root of the larger phylogeny [18]. Here we are using a single method of phylogenetic construction, but there are other methods that may alter PD estimates. However, recent analyses have shown that the method of phylogenetic construction does not appear to alter qualitative results [18], [38].

### Trait data

In the summers of 2007 and 2008, we measured leaf traits in the Cedar Creek biodiversity experiment [E120]. We sampled three fully mature leaves from ten individuals of each species collected within the maintained experimental plots as well as from the unmaintained experimental plots. Each individual was identified from a randomly chosen plot to cover the range of diversity treatments. We scanned fresh leaves on a flatbed scanner on the same day as collection with petioles and sheaths removed. Leaf area, perimeter and Feret's diameter (i.e., parallel lines touching opposites ends of the leaf) were calculated from the scanned leaves using ImageJ software [39]. These measures allowed calculation of perimeter per area (P/A, cm⋅cm^−2^), which is empirically correlated with leaf hydraulic conductance across a wide range of taxa [40]. Perimeter per leaf area⋅Feret's diameter is a unitless measure of leaf lobedness [e.g., 41] that influences the leaf radiation balance [42]. After scanning, we dried the leaves at 65°C for three days and weighed to calculate specific leaf area (cm^2^⋅g^−1^).

We determined seed mass by collecting seed heads for ten plants per species after seeds were fully mature, air drying the seeds, and then weighing together ten seeds (and dividing by ten) to calculate a mean seed mass per plant. For five species that were not seeding, seed mass was taken from online commercial databases, including the Native Seed Network (www.nativeseednetwork.org), Wildflower Farm Inc. (www.wildflowerfarm.com), and Prairie Moon Nursery (www.prairiemoon.com). Plant height was taken from the USDA Plants Database (plants.usda.gov). See table 1 for trait codes.

**Table 1:** Diversity variables calculated.

Variable	Code
Phylogenetic diversity	PD
Number of species	N
Number of functional groups	FG
Functional diversity (Petchey & Gaston 2002)	FD
Functional attribute diversity (Walker et al. 1999)	FAD
Functional diversity from non-metric multi dimensional scaling	NMDS
Variation in leaf area (SD)	LA
Variation in leaf perimeter area ratio (SD)	LPA
Variation in leaf lobiness (SD)	LL
Variation in specific leaf area (SD)	SLA
Variation in seed weight (SD)	SW
Variation in height (SD)	H
Presence of C3 grass	C3
Presence of C4 grass	C4
Presence of forb	F
Presence of N fixer	Nfix

**Figure 2:**
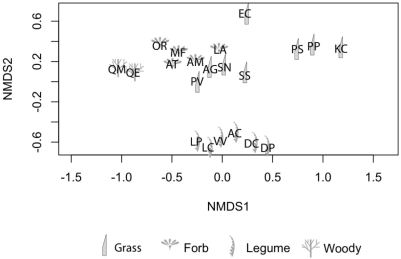
The ordination plot produced by nonmetric multi-dimensional scaling. Symbols refer to functional group membership.

Trait variation at the plot scale was estimated by the coefficient of variation in trait values for leaf area, leaf perimeter area ratio, leaf lobedness, specific leaf area, seed weight and height, while plot presence/absence was recorded for the C3, C4, forb and nitrogen fixer functional groups. We calculated four trait diversity metrics: the number of functional groups, FG; functional diversity, FD [24]; functional attribute diversity, FAD [23]; and Nonmetric Multi Dimensional Scaling, NMDS [e.g., 43]. FD estimates net species similarity or differences as branch lengths from a functional dendrogram based on a multivariate distance matrix [24]. To calculate FD, we scaled the traits to have a mean of zero and variance of one. We then calculated a Euclidean distance matrix and performed hierarchical clustering on this matrix and calculated FD as the total branch lengths connecting community members together [24]. For FAD, we again used the traits scaled to mean of zero and variance of 1 and calculated a Euclidean distance matrix. We then summed the distances for all species in a community [23]. Finally, since we were including multiple traits in these analyses, we wanted to account for groups of correlated traits. We performed NMDS on the trait matrix including functional groups (Fig. 2). We ran the analysis using two to five dimensions and choose the number of dimensions that reduced stress and which had no deviations on a dissimilarity-distance plot [44]. We selected a three-dimension model due to the stress reduction associate with this model (i.e., 8.71 versus 16.68 for the two-dimension model). We then calculated FD using the dimensions as independent traits. All variables are defined in Table 1. We have included the script, written in R 2.7.1 (www.R-project.org), to calculate PD, FD and FAD from a community membership list, a phylogeny and a trait matrix (see Appendix S2).

### Statistical analysis

There were a total of 16 different diversity variables used in this analysis (Table 1), and the goal was to construct the most informative model explaining patterns of biomass production. To narrow down the number of potential explanatory variables, we searched for the best single-variable models where average annual productivity was regressed against each diversity metric and compared to the explanatory ability of these models using Akaike weights, which can be interpreted as the probability that a particular model is the best fit to the observed data among a set of candidate models [45]. We further used mallow's *C_p_* to rank these single variable models. We checked diagnostic plots (e.g., residual versus fitted plots) for potential outliers and data trends.

We were not only interested in the best single variable explaining patterns of productivity, but also combining PD and trait metrics in multi-variable models. We performed a stratified all subsets model approach where PD was included in models with either single variable trait diversity metrics or multivariate functional diversity metrics, but not both since the multivariate metrics are based on the single variables. Mallow's *C_p_* was used to select potential models up to 5 variables and Akaike weights to compare them. Alternatively, we selected the best single variables and constructed models around these, comparing them using Akaike weights.

We also asked whether any of the continuous individual traits themselves had significant phylogenetic signals. To do this we generated phylogenetically independent contrasts [28] for the various traits. We compared the summed absolute node contrast values to that expected from 1000 randomizations (mean and 95% confidence intervals) [46]. If the observed summed contrast is significantly lower than the randomly generated values, sister clades tend to be more similar to one another than random, and there is a detectable phylogenetic signal. All analyses were done using R 2.7.1 (www.R-project.org)

## Results

**Figure 3:**
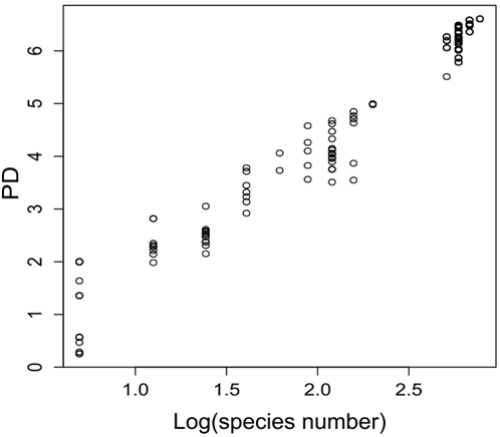
The relationship between species number and phylogenetic diversity.

**Figure 4:**
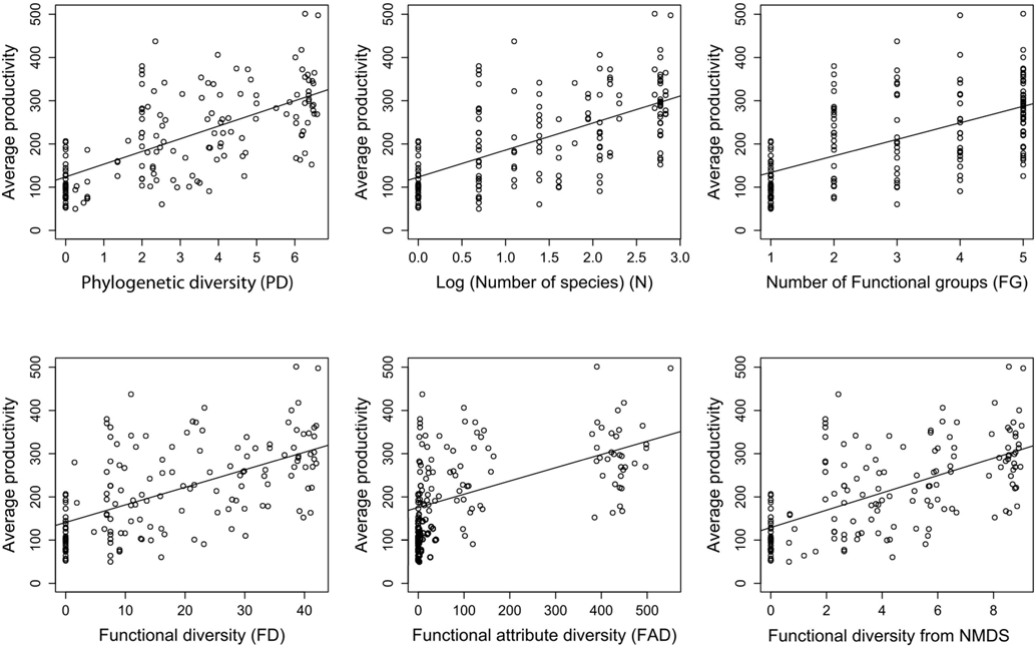
The relationship between average annual plot productivity and six diversity metrics. Of these six metrics, PD is the best single explanatory variable, second only to the presence of a nitrogen fixer (see Table 2).

Interestingly, the species in the FD dendrogram did not produce the same sister pairings as in the molecular phylogeny (Fig. 1), while the NMDS dendrogram shows species clustering more similar to the molecular phylogeny (Fig. 1). As would be expected because the number of branches, and thus the sum of all branch lengths, increases with the number of plant species (N), PD and N were highly correlated (Fig. 3). However, PD seems to better explain variation in productivity compared with N, FG, FD, FAD or NMDS (Table 2). Productivity was positively related to each of these measures (Fig. 4). The better fit provided by PD compared to the traditional variable, N, suggests that PD, which combines the effect of differences (via branch lengths) with the effect of the number of species (as N-1 branches) is a superior single measure of diversity than N.

**Table 2:** Results of univariate models.

Variable	Intercept	Slope	DF	Pvalue	AIC	R^2^	AW	Rank
Nfix	111.99	147.50	149	<0.001	1747.47	0.436	9.31×10^−01^	1
PD	123.76	29.37	149	<0.001	1752.78	0.415	6.54×10^−02^	2
SW	154.44	−5126.37	149	<0.001	1758.56	0.392	3.61×10^−03^	3
log(N)	123.30	62.51	149	<0.001	1764.20	0.369	2.16×10^−04^	4
NMDS	129.10	20.06	149	<0.001	1765.35	0.365	1.22×10^−04^	5
FG	95.62	38.29	149	<0.001	1771.54	0.338	5.51×10^−06^	6
FD	140.82	4.06	149	<0.001	1774.68	0.324	1.15×10^−06^	7
FAD	175.41	0.30	149	<0.001	1785.59	0.273	4.90×10^−09^	8
SLA	203.94	−4.02	149	<0.001	1817.14	0.105	6.90×10^−16^	9
F	171.70	66.04	149	<0.001	1818.94	0.094	2.81×10^−16^	10
C4	173.15	59.55	149	<0.001	1822.70	0.071	4.28×10^−17^	11
H	180.90	−203.85	149	0.002	1823.53	0.066	2.83×10^−17^	12
C3	181.58	51.87	149	0.003	1824.66	0.059	1.61×10^−17^	13
PA	214.95	−0.60	149	0.057	1830.13	0.024	1.04×10^−18^	14
LA	213.90	−0.24	149	0.253	1832.49	0.009	3.20×10^−19^	15
LPA	213.10	0.06	149	0.418	1833.15	0.004	2.30×10^−19^	16

AW is Akaikes weight which is the probability of model *I* being the best model explaining variation in average annual productivity. The presence of a nitrogen fixer was the best single variable model, followed by phylogenetic diversity, variation in seed weight, and log of the number of plant species. Rank indicates model ranking from Mallow's *C_p_*.

However, the single best explanatory variable was the presence of a nitrogen fixer (Table 2). Only within the plots with a nitrogen fixer present, which includes most plots, PD is still significantly related to productivity (F_1,103_ = 19.98, P<0.0001) and a better model (Akaike weight, AW = 0.634) than N, FAD, NMDS, FD or FG (AW = 0.142, 0.089, 0.084, 0.047 and 0.037, respectively).

While the above analyses informs our understanding of how individual models compare to one another, we were also interested in combining variables in models to better explain variation in productivity. We did this in two ways. First, we used a stratified all subsets approach (Table 3). The best models contained PD along with SW, PD and PA –while the multivariate functional diversity metrics were not selected as variables in the best models. Secondly, we took an informed approach by building various models from the top five predictors from Table 2. The best model of 26 potential models (AW≈1) is one that includes PD, SW and Nfix (Table S1).

**Table 3:** Comparison of multivariate predictor models from the stratified all subsets routine.

Variable	DF	Pvalue	AIC	R^2^	AW
PD	149	<0.001	1752.78	0.415	3.92×10^−20^
PD/Nfix	148	<0.001	1722.18	0.529	1.72×10^−13^
PD/SW	148	<0.001	1701.54	0.589	5.24×10^−09^
PD/SW/PA	147	<0.001	1683.04	0.641	5.43×10^−05^
PD/SW/Nfix	147	<0.001	1683.75	0.640	3.82×10^−05^
PD/SW/PA/Nfix	146	<0.001	1667.04	0.682	1.62×10^−01^
PD/SW/PA/LPA	146	<0.001	1680.46	0.652	1.98×10^−04^
PD/SW/PA/Nfix/LPA	145	<0.001	1664.53	0.691	5.68×10^−01^
PD/SW/PA/Nfix/FG	145	<0.001	1666.02	0.688	2.70×10^−01^

We also ran the model explicitly testing Westoby's (1998) LHS plant strategy scheme by regressing productivity against the additive effects of SLA, SW and H. While this model was significant (P<0.0001), only seed weight was significant as an individual model term (P<0.0001) while SLA and H were not (P = 0.261 and 0.190, respectively). Further, this model did not have much of an explanatory advantage over the model with seed weight alone (AIC = 1755.5 vs. 1756.7; and R^2^ = 0.41 vs. 0.40).

**Figure 5:**
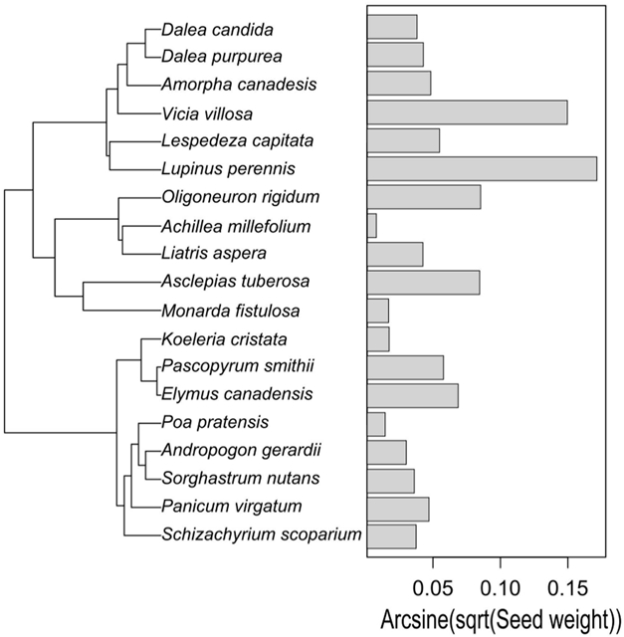
The distribution of seed size across the phylogeny.

For each of the six continuous traits, we assessed whether traits covaried with relatedness using phylogenetically independent contrasts. SW had a significant phylogenetic signal (summed observed contrasts = 0.131, null expectation [95% CI] = 0.185 [0.136–0.253]), while the rest of the traits lacked any significant signal (LA: obs = 192.5, null [95% CI] = 190.4 [149.5–235.2]; LPA: obs = 306.4, null [95% CI] = 301.8 [229.3–387.9]; LL: obs = 483.1, null [95% CI] = 293.5 [197.4–489.2]; SLA: obs = 1406.4, null [95% CI] = 1374.9 [1121.8–1693.4]; and H: obs = 14.8, null [95% CI] = 14.8 [11.9–18.1]). The significant signal in seed weight is influenced by the fact that species in the Fabaceae tended to have larger seed weights (Fig. 5). We confirmed that the non-Fabaceae species lacked a significant signal by removing Fabaceae species and re-running the analysis (obs = 0.042, null [95% CI] = 0.053 [0.037–0.067]), and species within the Fabaceae also lacked a signal (obs = 0.087, null [95% CI] = 0.104 [0.066–0.135]).

## Discussion

Phylogenetic diversity (PD) within a plot was an important factor explaining community productivity patterns. This result is not surprising; we fully expected PD to be a significant predictor of productivity, given that previous studies have shown this [e.g.], [9,18]. On the other hand, we expected multivariate functional diversity indices to better account for productivity patterns, since trait differences should drive ecological differences –regardless of patterns of shared ancestry on the traits. Even though multivariate functional diversity metrics were significant predictors of productivity patterns [see also 25], PD was by far a better predictor. Further, individual traits (besides nitrogen fixation and seed weight) did not show detectable phylogenetic signals, meaning that PD's influence on productivity is likely driven by other, unmeasured traits. This lack of signal could be due to the limited size and coverage of our phylogeny. Further, the seed weight signal appears driven by the fact that the species in the Fabaceae clade tend to have larger seeds than other species, meaning the seed weight results are at least partially driven by the covariance between seed size and being a nitrogen fixer. Although the alternative interpretation would be that the seed weight result likely represents the effect of some other traits involved in a tradeoff with seed size.

Beyond PD, the presence of a nitrogen fixer, was an important explanatory variable. Given that grassland communities at Cedar Creek are generally nitrogen-limited, it is not surprising that the presence of a nitrogen fixer was the single most important factor explaining patterns of community productivity [7]. Yet most polycultures contained at least one nitrogen fixer, meaning that the presence of nitrogen fixers does not inform our understanding of productivity patterns among the most speciose plots. Further, controlling for the presence of N-fixers allows us to overcome a potential phylogenetic “selection” effect. Thus the analyses examining the explanatory value of PD and the various trait measures in plots containing an N-fixer are particularly illuminating. Here again PD comes out as the best single measure, but we cannot eliminate the role of selecting other highly productive species or clades.

### Limitations and future directions

While the results presented here strongly support using PD to understand productivity patterns, there are at least two caveats that should be the focus of future research. First, our conclusion that PD is the best predictor of biomass production stems from a single study system at one spatial scale, and PD may not prove to be such a good predictor in other systems or at other scales. In a metaanalysis of PD effects on productivity across 29 experiments, Cadotte et al. (2008) found much variation in the explanatory value of PD (R^2^ ranged from 0.01 to 0.69). Further, in some systems or under some environmental conditions the critical functional traits may have much more important roles and thus more explanatory value than PD. Such critical traits may be very labile with a low phylogenetic signal, which a phylogeny fails to capture, or else these traits exhibit considerable phenotypic plasticity. Future analyses should include belowground traits, which may be critical in this system for understanding mechanisms of niche partitioning that enhance productivity. A mechanistic understanding of the productivity-diversity relationship may provide greater insight and is often more relevant for practical, management objectives than finding relationships that lack a clear mechanistic basis, as with PD.

Secondly, future research is also required to link PD to ecological differentiation. In this paper, we assume that change in PD is proportional to change in niche space or ecological function, meaning that the magnitude of variation in niche differentiation is dependent on the time since divergence (i.e., Brownian trait evolution). Under such a model, taxa covary in their niche trait based on the amount of time they are represented by a single ancestor in a phylogeny, then differentiate in that niche trait, according to a Markov process of probabilistic trait change diverging from some mean ancestral trait [27]. Other models relating phylogenetic distances to niche or phenotypic divergence could be compared, including those that do not assume that trait divergence is proportional to genetic change [47]. Further, alternative models have been proposed that incorporate explicit models of stabilizing or strong directional selection [e.g.], [48], [49,50]. Addressing alternative models of how phylogenetic distances correspond to trait changes would offer insight into what extent niches are conserved [51] and what this means for ecosystem function. Thus, future work should center on finding probable models that relate phylogenetic differentiation with actual niche and functional differences.

Given these caveats, it is still remarkable that PD is such a strong predictor of productivity patterns. PD might be further preferable because it is becoming increasingly simpler to measure PD compared with functional diversity metrics. Online databases, such as the GenBank (www.ncbi.nlm.nih.gov), the genetic sequence repository, or TreeBASE (www.treebase.org) that stores phylogenetic data from publications, or Phylomatic (www.phylodiversity.net/phylomatic/), which constructs phylogenies for species lists from available Angiosperm supertrees, provide straightforward paths to developing phylogenies. While trait repositories are being developed (e.g., TraitNet; www.columbia.edu/cu/traitnet/) there is no widely available, comprehensive source for multiple traits, and it is unlikely it would cover all the physical and biochemical traits that matter in different systems.

### Relating experimental results to natural communities

While phylogenetic diversity may be useful in understanding the consequences of diversity change in ecosystems not amenable to experimental manipulation (e.g., the open ocean, communities with endangered species, communities of long-lived organisms, etc.), future research needs to relate our results to mechanisms of coexistence and productivity in natural communities [10]. In a recent study by Flombaum and Sala [52], primary productivity in natural ecosystems showed a greater response to gradients in species number compared to those from artificially assembled experimental plots, such as with the data used here. Is the stronger diversity-productivity relationship in naturally assembled communities driven more by critical functional traits, or would PD still better explain productivity? Additionally, how communities are assembled could have important ramifications for patterns of PD. Natural community assembly does not select species randomly from a regional species pool, meaning that phylogenetic relationships or ecological traits may inform basic coexistence patterns. Phylogenetic relatedness likely depends on the spatial and taxonomic scale being considered [41], where at broad scales, phylogenetic clustering corresponds to trait selection, while at narrow scales, very close relatives are less likely to co-occur [41], [53]. Certain ecological traits should be selected for in the process of community assembly, and phylogenetic patterns likely reflect the phylogenetic signal of selected traits [54], [55]. Depending on the exact nature of trait evolution and community assembly, communities could show greater phylogenetic diversity with low trait variation and vice versa [55]. The relative contribution of trait variation vs. PD on ecosystem productivity may then also vary with community assembly processes; for instance if community assembly favors high PD and lower critical trait variance [55], then PD may still represent other ecological traits, and thus be significantly related to productivity. Phylogenetic information may be a way to scale from organismal physiology to ecosystem-level processes [56], [57].

### Conclusions and implications

We explicitly tested the explanatory power of PD on community productivity against a suite of individual and multivariate traits for experimentally created grassland communities. The trait metrics did not explain productivity patterns as well as PD and one major reason appears to be that we did not measure those functional traits, such as root types, rooting depth or resource requirements, which the phylogeny is representing. We assume that since PD is such a good explanatory variable, it provides a measure of diversity that must be associated with critical functional differences among species within a community that contribute to maximization of productivity.

Even without a clear mechanistic basis, these results have important implications for habitat restoration and biofuel production. If one of the goals of habitat restoration activities is to maximize community functionality, then restoration biologists should attempt to maximize the evolutionary diversity among members of the planned community. For biofuel production, especially in Midwestern prairies, in addition to choosing species with fast growth rates, biofuel-producing communities should consist of phylogenetically distinct mixtures while paying attention to critical functional groups such as nitrogen fixers.

## Supporting Information

Appendix S1 Table of species, genes and GenBank accession numbers and the phylogeny.
(0.81 MB DOC)

Appendix S2 R scripts to calculate PD and various measures of trait diversity.
(0.04 MB DOC)

Table S1 Multiple model comparison.
(0.06 MB DOC)
