# Supplementary material for: Using Phylogenetic, Functional and Trait Diversity to Understand Patterns of Plant Community Productivity
Source: PLoS One. 2009 May 27;4(5):e5695. doi: 10.1371/journal.pone.0005695 (PMC2682649; doi:10.1371/journal.pone.0005695)
Supplement: Appendix S1 — Table of species, genes and GenBank accession numbers and the phylogeny. (0.81 MB DOC) [file pone.0005695.s001.doc]

Appendix S1: *Table of species, genes and GenBank accession numbers and the phylogeny.*

| Taxa | Exp | *rbcl* | *matK* | *5.8S* | *ITS1* | Congen |
| --- | --- | --- | --- | --- | --- | --- |
| *Achillea millefolium* | Both | EU384938 | n | EU796891 | n | n |
| *Amorpha canadesis* | 120 | U74212 | AY391785 | AY426774 | AY426774 | *Amorpha fruticosa* |
| *Andropogon gerardii* | Both | AJ784818 | AF144577 | n | n | n |
| *Anemone cylindrica* | 123 | n | AB110531 | n | AB120213 | *Anemone cernua* |
| *Asclepias tuberosa* | Both | EF590504 | n | EF590756 | n | n |
| *Astragalus canadensis* | 123 | n | AY386875 | n | L10770 | n |
| *Bouteloua gracilis* | 123 | AJ784829 | n | EU144388 | EU144388 | n |
| *Buchloe dactyloides* | 123 | n | AF312325 | n | n | n |
| *Coreopsis palmata* | 123 | L13642 | AY551493 | n | AY553707 | *Coreopsis grandiflora* |
| *Dalea candida* | 120 | n | AY386860 | n | AY426793 | *Dalea pulchra* |
| *Dalea purpurea* | Both | n | AY391798 | n | AY426794 | n |
| *Elymus canadensis* | Both | AM849350 | n | n | n | *Elymus farctus* |
| *Euphorbia pubentissima* | 123 | AB233884 | AB233780 | n | n | *Euphorbia humifusa* |
| *Koeleria cristata* | Both | AJ784825 | n | n | DQ336827 | *Koeleria pyramidata* |
| *Lespedeza capitata* | Both | U74215 | n | n | n | *Lespedeza cuneata* |
| *Liatris aspera* | Both | AY816235 | n | n | AY804146 | *Liatris cylindracea* |
| *Lupinus perennis* | 120 | Z70066 | n | DQ524249 | DQ524249 | *Lupinus luteus* |
| *Monarda fistulosa* | 120 | Z37419 | n | AY943493 | n | n |
| *Oligoneuron rigidum* | 120 | n | n | AY523851 | AY523851 | n |
| *Panicum virgatum* | Both | AY632368 | n | DQ005062 | DQ005062 | n |
| *Pascopyrum smithii* | Both | AY836156 | n | n | AY740890 | *Agropyron cristatum* |
| *Poa pratensis* | Both | n | AM234593 | AB127955 | n | *Poa annua* |
| *Quercus ellipsoidalis* | 120 | n | EU749366 | AF098418 | n | *Quercus rubra* |
| *Quercus macrocarpa* | 120 | n | n | AF098419 | n | *Quercus alba* |
| *Rudbeckia hirta* | 123 | n | n | n | n | n |
| *Schizachyrium scoparium* | Both | n | n | DQ005072 | DQ005072 | n |
| *Solidago nemoralis* | 123 | DQ006067 | n | EU125362 | EU125362 | *Solidago gigantea* |
| *Sorghastrum nutans* | Both | EF125121 | n | n | n | n |
| *Sporobolus cryptandrus* | 123 | n | AF144601 | EU646110 | EU646110 | *Sporobolus indicus* |
| *Symphyotrichum oolentangiense* | 123 | n | EU749439 | n | EU200229 | n |
| *Vicia villosa* | 123 | n | AF522161 | DQ312199 | DQ312199 | n |
| **Additional taxa** |  |  |  |  |  |  |
| *Amborella trichopoda* |  | L12628 | NC_005086 | n | n |  |
| *Magnolia grandiflora* |  | EF590545 | EF590407 | n | n |  |
| *Lactuca sativa* |  | AP007232 | AP007232 | AM411184 | AM411184 |  |
| *Asclepias incarnata* |  | DQ006053 | n | DQ005967 | n |  |
| *Salvia officinalis* |  | AY570431 | n | n | n |  |
| *Quercus serrata* |  | AB060576 | AB060067 | n | n |  |

((Magnolia_grandiflora:0.04749,Amborella_trichopoda:0.141321)0.877000:0.0080285,((((Bouteloua_gracilis:0.055299,Schizachyrium_scoparium:0.179071)0.058000:0.013591,(Panicum_virgatum:0.043921,((Sorghastrum_nutans:0.001392,Andropogon_gerardii:0.004429)0.826000:0.009624,((Sporobolus_cryptandrus:0.043752,Buchloe_dactyloides:0.030027)0.863000:0.01107,Poa_pratensis:0.096489)0.951000:0.018588)0.947000:0.024782)0.953000:0.022698)0.925000:0.029753,((Elymus_canadensis:0.00552,Pascopyrum_smithii:0.010382)0.988000:0.030761,Koeleria_cristata:0.023313)0.921000:0.039637)1.000000:0.268846,(Anemone_cylindrica:0.180639,((((Salvia_officinalis:0.005257,Monarda_fistulosa:0.012795)0.974000:0.051126,(Asclepias_tuberosa:0.065927,Asclepias_incarnata:0.002697)1.000000:0.189149)0.942000:0.061359,((((Liatris_aspera:0.064393,Coreopsis_palmata:0.037974,Rudbeckia_hirta:0.05118)0.998000:0.025406,(Achillea_millefolium:0.043562,Lactuca_sativa:0.028824)0.956000:0.023085)0.807000:0.007382,(Symphyotrichum_oolentangiense:0.054735,(Solidago_nemoralis:0.0,Oligoneuron_rigidum:0.00162)0.927000:0.015239)0.968000:0.028916)0.777000:0.006146,Symphyotrichum_cordifolium:0.020208)1.000000:0.077771)0.843000:0.04181,(Euphorbia_pubentissima:0.125511,(((Quercus_serrata:0.002244,Quercus_ellipsoidalis:0.0068)0.765000:0.036008,Quercus_macrocarpa:0.0)0.906000:0.077946,((Lupinus_perennis:0.058336,Lespedeza_capitata:0.113526)0.306000:0.005766,((Vicia_villosa:0.074364,Astragalus_canadensis:0.045926)1.000000:0.04969,(Amorpha_canadesis:0.023175,(Dalea_purpurea:0.05049,Dalea_candida:0.055157):0.05678)1.000000:0.019707)0.993000:0.02651)0.946000:0.073925)1.000000:0.015029)0.740000:0.037682)0.999000:0.039026)0.988000:0.019496)0.922000:0.0080285);


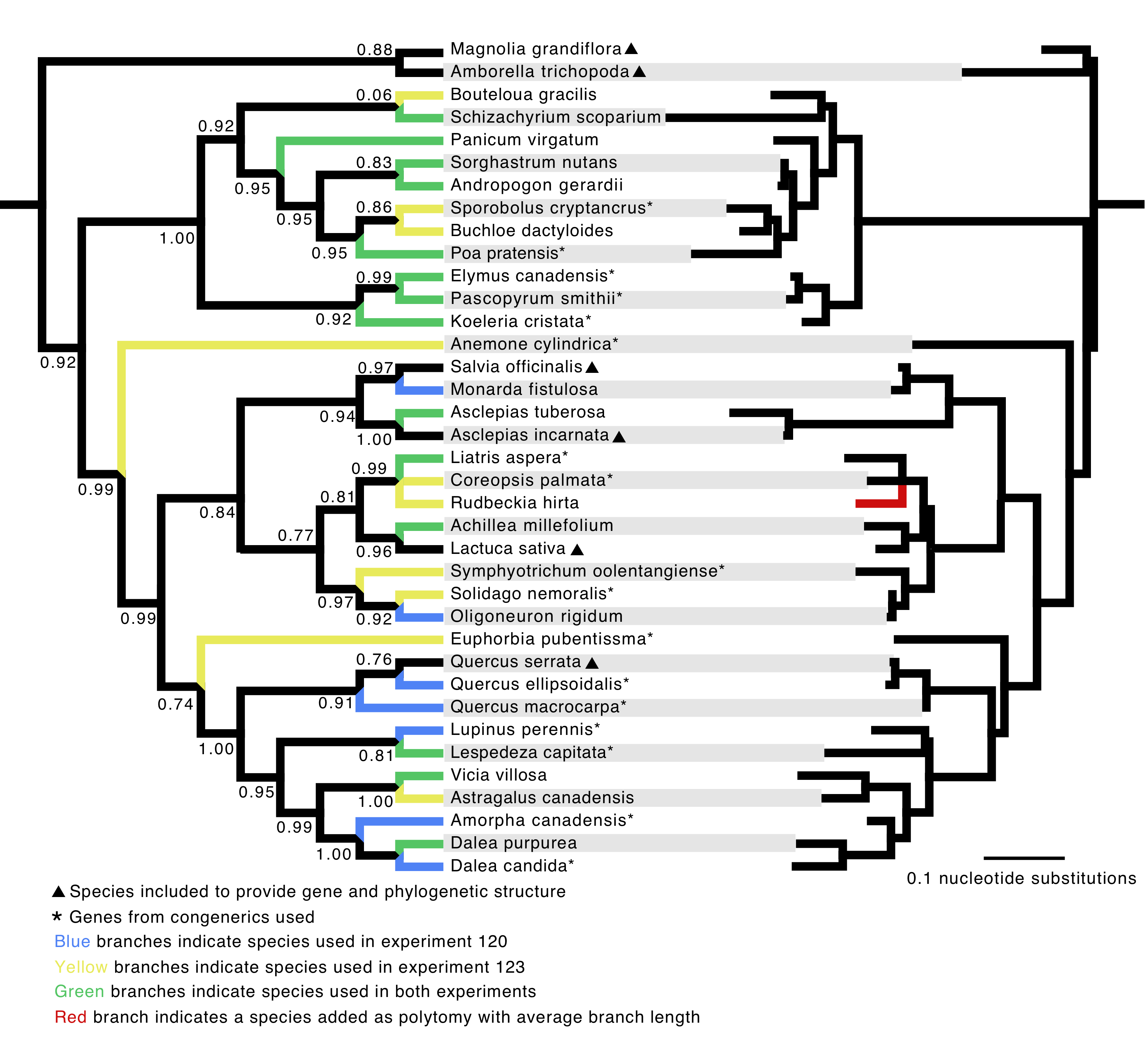


**Figure S1-1**: The full phylogeny for Cedar Creek experiments 120 and 123
