# Supplementary material for: Using Phylogenetic, Functional and Trait Diversity to Understand Patterns of Plant Community Productivity
Source: PLoS One. 2009 May 27;4(5):e5695. doi: 10.1371/journal.pone.0005695 (PMC2682649; doi:10.1371/journal.pone.0005695)
Supplement: Appendix S2 — R scripts to calculate PD and various measures of trait diversity. (0.04 MB DOC) [file pone.0005695.s002.doc]

Appendix S2: *R scripts to calculate PD and various measures of trait diversity.*

#####script to calculate several community diversity metrics from phylogenetic, trait and community composition datasets.

####Written by Marc W Cadotte

####July 2008

#Requires ape package

library(ape)

#phylogeny file (newick format), use read.nexus for nexus format file

my.tree<-read.tree("file name")

#this is file with 2 columns with NO headers. Column 1 is species names and col 2 is community names (e.g., 1, 2... or A, B ..., etc.)

sp.list<-read.table("file name", header=F)

#this is a trait matrix, where col 1 is species names and col 2...n are various continuous traits (or 1 & 0 for discrete states). This file needs headers. Make sure species names in all three files are identical, use the "match" command to find errors.

trait.data<-read.table(file="file name", header=T)

#make tree ultrametric if needed

if (!is.ultrametric(my.tree)) ultra.tree<-chronogram(my.tree)

species<-as.list(ultra.tree$tip.label)#list of species in tree

traits<-scale(trait.data[,2:ncol(trait.data)]) #standardize trait data to mean = 0 and var = 1

trait.clust<-hclust(dist(traits), method="average") #hierarchical clustering as per Petchey & Gaston

trait.clust$labels<-species[trait.clust$order] #label cluster objects as species names -plot by "plot(trait.clust)"

clust.phy <- as.phylo(trait.clust) #transform trait.clust into phylo object

##NMDS ordination of traits. Need to do sensitivity analysis to select optimal number of dimensions (i.e., minimizing stress), in the example here I use 3 dimensions (k = 3)

trait.mds<-metaMDS(trait.data[,2:ncol(trait.data)], distance="jaccard", k = 3, trace=FALSE)

rownames(trait.mds$points)<-trait.data$species

mds.clust<-hclust(dist(trait.mds$points), method="average")

mds.phy <- as.phylo(mds.clust)

comID<-unique(sp.list[,2]) #community names

#for output

PD<-numeric(length(comID)) #phylogenetic diversity

sp.rich<-numeric(length(comID)) #number of species

FD<-numeric(length(comID)) #Functional diversity (Petchey & Gaston 2002)

FAD<-numeric(length(comID)) # Functional attribute diversity (Walker et al. 1999)

CV.D<-data.frame()#dataframe for single variable standard deviations

for (i in 1:length(comID)) {#loop through community IDs & calculate PD, FD and FAD

ID<-comID[i]#select community.ID

myclade<-sp.list[sp.list[,2]==ID,]#get list of species in that community

dropme<-species[!species %in% myclade[,1]]#species in tree but not in community

sub.tree<-drop.tip(ultra.tree, dropme) #create phylogeny for individual community

PD[i]<-sum(sub.tree$edge.length) #calculate phylogenetic diversity

sp.rich[i]<-length(sub.tree$tip.label) #number of species

sub.clust<-drop.tip(clust.phy,dropme) #subset of trait dendrogram

FD[i] <- sum(sub.clust$edge.length) #calculate functional diversity

fad.spp<-match(myclade[,1], trait.data$species)

sub.traits<-traits[fad.spp,]

FAD[i] <-sum(dist(sub.traits))

sub.mds<-drop.tip(mds.phy, dropme) #subset of mds dendrogram

MDS[i] <- sum(sub.mds$edge.length) #calculate functional diversity

CV<-matrix(nrow=1, ncol=ncol(traits)) #temporarily hold CV values

for (j in 1:ncol(traits)) {#loop to calculate CV values for each trait in community i

CV[,j] <- sd(sub.traits[,j])/mean(sub.traits[,j]) #calculate CV and add it to temp holder

} #end j loop

CV.D<-data.frame(rbind(CV.D, as.data.frame(CV))) #add temp holder to perm dataframe

}#end i loop

colnames(CV.D)<-colnames(traits)

sp.rich<-log(sp.rich)

#OUTPUT RESULTS

results <- data.frame(comID, PD, sp.rich, FD, FAD, MDS)

results <- data.frame(cbind(results,CV.D))

write.csv(results, file="output file name")

####function to calculate the sum of absolute phylogentically independent contrasts values and compare them to null values. Requires the package "ape". Arguments: dat is a vector of values sorted according to species in phylogeny, tree is the phylogeny and n.rand is the number of randomizations. Example sorting: dat<-trait.data$trait1[match(tree$tip.label,trait.data$species)]

pic.func<-function(dat,tree,n.rand=1000){

pic.null<-NULL

for (i in 1:n.rand){

tmp<-sample(dat,size=length(dat))

pic.null[i]<-sum(abs(pic(tmp,tree)))

}

results<-data.frame(pic.obs=sum(abs(pic(dat,tree))),

pic.null.mean=mean(pic.null),

pic.null.05=quantile(pic.null,probs=c(0.05,0.95))[1],

pic.null.95=quantile(pic.null,probs=c(0.05,0.95))[2],

row.names=NULL

)

return(results)

}
