# Supplementary material for: Using Phylogenetic, Functional and Trait Diversity to Understand Patterns of Plant Community Productivity
Source: PLoS One. 2009 May 27;4(5):e5695. doi: 10.1371/journal.pone.0005695 (PMC2682649; doi:10.1371/journal.pone.0005695)
Supplement: Table S1 — Multiple model comparison. (0.06 MB DOC) [file pone.0005695.s003.doc]

**Table S1:** Comparison of multivariate predictor models. The best model is highlighted in bold.

| Model | DF | AIC | R^2^ | AW |
| --- | --- | --- | --- | --- |
| *Prod* = *β_0_* + *β_1_⋅PD* | 149 | 1752.78 | 0.415 | 3.54E-17 |
| *Prod* = *β_0_* + *β_1_⋅Nfix* | 149 | 1747.47 | 0.436 | 5.03E-16 |
| *Prod* = *β_0_* + *β_1_⋅PD* + *β_2_⋅Log(N)* + *β_12_⋅PD⋅ Log(N)* | 147 | 1747.73 | 0.449 | 4.40E-16 |
| *Prod* = *β_0_* + *β_1_⋅PD* + *β_2_⋅ Log(N)* | 148 | 1750.44 | 0.432 | 1.14E-16 |
| *Prod* = *β_0_* + *β_1_⋅PD* + *β_2_⋅MDS* + *β_12_⋅PD⋅MDS* | 147 | 1751.71 | 0.435 | 6.01E-17 |
| *Prod* = *β_0_* + *β_1_⋅PD* + *β_2_⋅MDS* | 148 | 1750.97 | 0.430 | 8.72E-17 |
| *Prod* = *β_0_* + *β_1_⋅PD* + *β_2_⋅ Log(N)* + *β_3_⋅MDS* + *β_12_⋅PD⋅ Log(N)* + *β_13_⋅PD⋅* *MDS* + *β_23_⋅ Log(N)⋅MDS* | 144 | 1750.37 | 0.461 | 1.18E-16 |
| *Prod* = *β_0_* + *β_1_⋅PD* + *β_2_⋅ Log(N)* + *β_3_⋅MDS* | 147 | 1751.85 | 0.434 | 5.62E-17 |
| *Prod* = *β_0_* + *β_1_⋅PD* + *β_2_⋅FD* + *β_12_⋅PD⋅FD* | 147 | 1746.93 | 0.452 | 6.57E-16 |
| *Prod* = *β_0_* + *β_1_⋅PD* + *β_2_⋅FD* | 148 | 1744.93 | 0.452 | 1.78E-15 |
| *Prod* = *β_0_* + *β_1_⋅PD* + *β_2_⋅ Log(N)* + *β_3_⋅FD* + *β_12_⋅PD⋅ Log(N)* + *β_13_⋅PD⋅* *FD* + *β_23_⋅ Log(N)⋅FD* | 144 | 1746.77 | 0.474 | 7.11E-16 |
| *Prod* = *β_0_* + *β_1_⋅PD* + *β_2_⋅ Log(N)* + *β_3_⋅FD* | 147 | 1746.58 | 0.454 | 7.84E-16 |
| *Prod* = *β_0_* + *β_1_⋅Nfix* + *β_2_⋅ Log(N)* + *β_12_⋅Nfix⋅ Log(N)* | 147 | 1728.51 | 0.515 | 6.58E-12 |
| *Prod* = *β_0_* + *β_1_⋅Nfix* + *β_2_⋅ Log(N)* | 148 | 1726.56 | 0.515 | 1.74E-11 |
| *Prod* = *β_0_* + *β_1_⋅Nfix* + *β_2_⋅MDS* + *β_12_⋅Nfix⋅MDS* | 147 | 1729.52 | 0.512 | 3.96E-12 |
| *Prod* = *β_0_* + *β_1_⋅Nfix* + *β_2_⋅MDS* | 148 | 1727.53 | 0.512 | 1.07E-11 |
| *Prod* = *β_0_* + *β_1_⋅PD* + *β_2_⋅ Log(N)* + *β_3_⋅Nfix* + *β_12_⋅PD⋅ Log(N)* + *β_13_⋅PD⋅* *Nfix* + *β_23_⋅ Log(N)⋅Nfix* | 144 | 1726.67 | 0.540 | 1.65E-11 |
| *Prod* = *β_0_* + *β_1_⋅PD* + *β_2_⋅ Log(N)* + *β_3_⋅Nfix* | 147 | 1722.73 | 0.533 | 1.18E-10 |
| *Prod* = *β_0_* + *β_1_⋅PD* + *β_2_⋅Nfix* + *β_12_⋅PD⋅Nfix* | 147 | 1724.18 | 0.529 | 5.71E-11 |
| *Prod* = *β_0_* + *β_1_⋅PD* + *β_2_⋅ Log(N)* + *β_3_⋅Nfix* | 148 | 1722.19 | 0.529 | 1.55E-10 |
| ***Prod* = *β_0_* + *β_1_⋅PD* + *β_2_⋅SW^2^* + *β_3_⋅Nfix* + *β_12_⋅PD⋅SW^2^* + *β_13_⋅PD⋅* *Nfix* + *β_23_⋅SW^2^⋅Nfix*** | **144** | **1677.02** | **0.669** | **9.99E-01** |
| *Prod* = *β_0_* + *β_1_⋅PD* + *β_2_⋅SW* + *β_3_⋅Nfix* | 147 | 1690.79 | 0.622 | 1.02E-03 |
| *Prod* = *β_0_* + *β_1_⋅PD* + *β_2_⋅H* + *β_3_⋅Nfix* + *β_12_⋅PD⋅H* + *β_13_⋅PD⋅* *Nfix* + *β_23_⋅H⋅Nfix* | 144 | 1724.39 | 0.547 | 5.16E-11 |
| *Prod* = *β_0_* + *β_1_⋅PD* + *β_2_⋅H* + *β_3_⋅Nfix* | 147 | 1723.30 | 0.532 | 8.88E-11 |
| *Prod* = *β_0_* + *β_1_⋅PD* + *β_2_⋅H* + *β_3_⋅Nfix* + *β_4_⋅SW^2^* + *β_12_⋅PD⋅H* + *β_13_⋅PD⋅* *Nfix* + *β_14_⋅PD⋅SW^2^* + *β_23_⋅H⋅Nfix* + *β_24_⋅H⋅SW^2^* + *β_34_⋅Nfix⋅SW^2^* | 140 | 1723.30 | 0.690 | 8.88E-11 |
| *Prod* = *β_0_* + *β_1_⋅PD* + *β_2_⋅H* + *β_3_⋅Nfix + β_3_⋅SW* | 146 | 1692.74 | 0.622 | 3.85E-04 |
